# Agent-Based Modeling of the Immune System: NetLogo, a Promising Framework

**DOI:** 10.1155/2014/907171

**Published:** 2014-04-22

**Authors:** Ferdinando Chiacchio, Marzio Pennisi, Giulia Russo, Santo Motta, Francesco Pappalardo

**Affiliations:** ^1^Department of Electric, Electronics and Computer Engineering, University of Catania, V.le A. Doria 6, 95125 Catania, Italy; ^2^Department of Mathematics and Computer Science, University of Catania, V.le A. Doria 6, 95125 Catania, Italy; ^3^Department of Drug Sciences, University of Catania, V.le A. Doria 6, 95125 Catania, Italy

## Abstract

Several components that interact with each other to evolve a complex, and, in some cases, unexpected behavior, represents one of the main and fascinating features of the mammalian immune system. Agent-based modeling and cellular automata belong to a class of discrete mathematical approaches in which entities (agents) sense local information and undertake actions over time according to predefined rules. The strength of this approach is characterized by the appearance of a global behavior that emerges from interactions among agents. This behavior is unpredictable, as it does not follow linear rules. There are a lot of works that investigates the immune system with agent-based modeling and cellular automata. They have shown the ability to see clearly and intuitively into the nature of immunological processes. NetLogo is a multiagent programming language and modeling environment for simulating complex phenomena. It is designed for both research and education and is used across a wide range of disciplines and education levels. In this paper, we summarize NetLogo applications to immunology and, particularly, how this framework can help in the development and formulation of hypotheses that might drive further experimental investigations of disease mechanisms.

## 1. Introduction

Complex biological scenarios have been recently investigated with the synergic union between computational modeling and high-throughput experimental data. This approach has helped the generation of novel insights and hypotheses for further research and development, with a considerable saving in terms of time and costs. Moreover, it allowed experiments and/or measurements that cannot be easily achievable in a laboratory environment [1].

Once developed and validated, models can be adapted in different ways (e.g., inputs can be altered to mimic different environments) to enable examination of different qualities. These in silico (or dry-laboratory) experiments are of course complementary to traditional wet-laboratory experimental approaches [2].

During the last decades many mathematical and computational models have been developed to model and describe the immune system processes and features. Nevertheless it is possible to group most models in two large classes according to the modeling approach used: Top-down and bottom-up approaches (see [3]).

The Top-down approach works by estimating the mean behavior at a macroscopic level, thus modeling populations and not single entities. By using such an approach it is possible to model and represent a large number of entities. The oldest and most famous top-down approach has been represented by the use of ordinary and partial differential equations- based (ODE and PDE) models. Usually ODE models ignore the topology of the problem, whereas PDE can also be used when the space distribution is of importance for the problem. Both techniques neglect individual interactions. Models based on these approaches rely on a strong mathematical theory that allows in some cases analytical study and asymptotic analysis. However complex problems may entitle intractable models, and approximations of the biological scenario become a prerogative. Examples of models based on these approaches are presented in [4–8]. This class of models also includes stochastic differential equations, which add to the classical definition of differential equation some stochastic terms in order to mimic, for example, individual diversity or environmental fluctuations due to statistical noise (see, e.g., [9]).

The bottom-up approach works at a microscopic level. Entities (*agents*) and interactions are described and followed individually, and the general behavior of the system arises from the sum of the local behaviors of the involved entities. In this way it is possible to describe local immunological processes with greater accuracy, avoiding rough approximations that are typical of top-down approaches. Furthermore spatial distribution and stochastic behaviors generally are already inside, since they normally represent two intrinsic features of the modeling techniques that are based on a bottom-up approach. Conversely, since entities are followed individually, such modeling techniques require a bigger computational effort and there are no strong mathematical instruments that can permit any analytical study. Cellular automata and (multi-) agent-based methods are the most used bottom-up approaches, also in immunology [10–18].

Cellular automata and multiagent-based models have been initially developed for modeling specific problems by using general purpose programming languages, mainly C (in all of its variants) or Fortran. Soon after that the strength of these modeling techniques became clear to the scientific community; many agent-based languages and frameworks that enabled creating agent-based applications were developed (see [19] for a review).

Among these NetLogo, a programming language and integrated modeling suite that is totally devoted to ABMs, has reached a good level of maturity and usability. NetLogo development was started in 1999 by Uri Wilensky, which continues to maintain, update, and add new functions ever since [20, 21].

NetLogo is a functional programming language [22] with “turtles” that represent the agents and “patches” that represent a given point into the simulation space. Both of these may have multiple properties that can be defined by the user such as age, color, and position.

In practice, the fact that NetLogo uses a functional programming language means that many language statements are almost read as sentences, and this enables even unskilled and untrained users to understand and learn it through the examples.

NetLogo can be slower than other tools, but it is very easy to use, it supports the automatic drawing of agents in 2D or 3D, it gives the possibility to simply build user interfaces, and it is supplied with a lot of examples and HOWTOs, making it a suitable platform for beginner programmers. Moreover, NetLogo models can be effortlessly shared as Java applets, and this means that such models can be run in almost all (if not all) computer platforms. It is also possible to perform better statistical analysis of results thanks to a plug-in that allows communication between NetLogo and R [23], a very well-known statistical software. NetLogo has been released under the GNU General Public License, meaning that it is free and open-source. NetLogo thus represents a good choice to realize multiagents models, networks, and complex dynamical systems.

In this review we will focus on the use of NetLogo to model the immune system processes and features.

## 2. Application of NetLogo to Immunology

NetLogo framework has been used extensively to further our understanding of systems in several different disciplines, including biology, ecology, economics, and sociology. Here we will show several applications of NetLogo to model, at different extent, immune system dynamics.

### 2.1. Modeling Innate Immunity

Toll-like receptors (TLRs) represent a class of proteins playing an important function in the innate immune system. They are receptors usually expressed in macrophages and dendritic cells that recognize structurally conserved molecules derived from microbes. They are fundamental in activating the innate immune system response. In [24], the authors present spatially configured stochastic reaction chambers (SCSRC), that is, an agent-based modeling framework that incorporates an abstracted molecular “event” rule system with a spatially explicit representation of the relationship between signaling and synthetic compounds. They implemented the SCSRC with NetLogo and applied the model to TLR 4 signaling and the inflammatory response.

The model was able to accurately reproduce the dynamics of TLR-4 signaling in response to LPS stimulation. In particular, it was capable of showing that there was a dose dependent proinflammatory response effect and also the establishment of tolerance.

Inflammation is part of the first immune response, typically innate immune response to harmful stimuli, such as pathogens, damaged cells, or irritants. There are two main types of inflammation: acute or chronic. The former is the initial response of the body to dangerous stimuli and is sustained by the increased movement of plasma and leukocytes (especially granulocytes) from the blood into the injured tissues. The former is represented by a protracted inflammation, characterized by both destruction and healing of the tissue involved in the inflammatory process.

Brown et al. [25] developed a model to study the response of inflammatory cells (macrophages) and cells involved in remodeling (fibroblasts) to particulates exposure. The NetLogo model, even with clear limitations (varied tissue structures in the lung, missing full complement of cells, and other important immune cells that play a role in the inflammatory process), was able to forecast the existence of biologically relevant aspects, that is, healing and return to baseline, localized tissue damage and fibrosis, and extensive damage and fibrosis. It includes interactions among macrophages (that could be considered along with other lymphocytes and neutrophils, the leaders of inflammatory response), fibroblasts (that can lead to fibrosis), proinflammatory cytokine (TNF-a), anti-inflammatory cytokine (TGF-b1), collagen deposition, and tissue damage/dysfunction/DAMPs.

Another example of inflammation modeling is given in [26]. Here the authors develop an ABM of the epithelium using results derived from an in vitro model of gut epithelial permeability. Then, the epithelial ABM was integrated with the endothelial/inflammatory cell ABM to produce an organ model of the gut. This is an interesting example where the model results were then validated against in vivo models of the inflammatory response of the gut to ischemia.

A particular form of inflammation is characterized from the acute inflammatory response that arises initially in response to several biological stressors, including infection, burns, trauma, and invasive surgery. Under normal circumstances this kind of inflammation is strictly supervised by the immune systems and is regulated and self-limited. However when anti-inflammatory processes fail, one can observe an amplified inflammatory state that is depicted by severe, uncontrolled systemic inflammation and multiple organ dysfunction. In [27], Dong et al. proposed an ABM framework developed to investigate insight on the stochastic interactions of the mediators involved in the propagation of endotoxin signaling at the cellular response level that at the end triggers acute inflammation.

### 2.2. Modeling Immunity to Pathogens

Human papillomavirus (HPV) is a DNA virus from the papillomavirus family that is capable of infecting humans. The main targets of HPV are the keratinocytes of the skin or mucous membranes. Some types of infections can trigger benign papillomas, while others can lead to cancers of the cervix, vulva, vagina, penis, oropharynx, and anus [28]. NetLogo can interact with other modeling strategies and in [29] the authors combined cellular automata and agent-based modeling techniques (implemented in NetLogo) to simulate the growth and the HPV life cycle, allowing the observations at different stages.

In the adaptive immune response to a virus of an intracellular bacterium, the presentation of the antigen on the surface of the infected cell is of extreme importance. One way to perform this communication strategy is represented by the displaying of viral antigen on major histocompatibility complex of class I of the infected cell. Recently, another mode of communication has been revealed, namely, the transport of antigen from one cell to another through gap junctions [30]. Gap junctions are small channels that can form between two cells to permit the transfer of small molecules [31]. One of the advantages of NetLogo is to have available a graphical support without worrying about programming it. This permits having one of the main features of ABM ready to use: spatiality. A work that models gap junctions both with ABM and ODE techniques can be found in [32]. Here the authors showed by an ODE model that gap junction-mediated antigen transport is not a good strategy for the immune response in the situation where the infection dynamics can be explained by a well-mixed model (this can apply to organs, e.g., the spleen). In different situations, that is, where target cells are sparse and unmoving (this can represent, e.g., the case of epithelial cells that are targeted by many viruses), gap junction-mediated antigen transport might drive to a phenomenon similar to firebreaks, which impedes virus diffusion and can therefore establish a useful immune response scheme. This was modeled using NetLogo to implement a localized infection of a patch of epithelial tissue containing more than 10000 target cells on a grid of size 101 × 101. It is worth mentioning that in this case spatiality was of main importance.

### 2.3. Modeling Immune System Dynamics

Developing and using mathematical and simulation strategies cover also an important role in the analysis of the dynamics of infectious diseases in populations. Recently, such models integrate population structure and viewed differences between individuals in traits that impact transmission [33]. In this scenario, there are several cases in which the interaction between trait and infection dynamics plays an important role. Think, for example, about the systems where the heterogeneity between individuals affects implicit trait, that is, “infection history,” and the more explicit trait, that is, “immune status.” Patterns of such systems are mostly host-parasite systems with protozoan parasites or worm parasites. In [34], the authors use NetLogo to implement a model to analyze the host dynamics of a protozoan parasite infecting chickens, including acquired immunity and repeated infection through a spatially structured environment. The choice to use an individual-based model allowed the authors to investigate stochastically emerging heterogeneity. Main emerging results were the possibility to examine the variation in the immune response, transmission, and movement of individuals.

Understanding the role of leukocyte trafficking through the microcirculation and into tissues is of fundamental importance in vascular-associated pathologies like atherosclerosis, stroke, chronic wounds, and peripheral vascular diseases. In particular, methodologies that explore leukocyte adhesion and its dynamics are central. A computational framework that combines agent-based modeling (ABM) with a network flow analysis to study monocyte homing is presented in [35]. Here the authors show an important feature of NetLogo environment, that is, to import a specific map or network in which the agents are free to move. This can be of any type and can contain constraints (densities, barriers, one way paths, etc.). The paper describes a network blood flow model that computes fluid flow velocities, pressure distributions, and WSS values in a simulated microvascular network, taking the inputs from the ABM drawing program in NetLogo. The computational model was able to capture the multicell tissue-level dynamics of monocyte trafficking within a microvascular network bed. Thanks to the spatial representation ability of ABM, one of the interesting features of the model implemented in this paper is the possibility to follow the cells simulated both in space and time, which is currently not possible in experimental models.

### 2.4. Modeling Diseases

Multiple sclerosis (MS) is an inflammatory disease that affects the brain and spinal cord. MS is considered a CD4+ Th1-mediated autoimmune disease [36]. A NetLogo framework, that describes the typical scenario observed in relapsing-remitting multiple sclerosis (the most relevant type of MS), is presented in [37]. The main hypothesis of the model is given by the fact that the arising of the pathology is due to a breakdown in the peripheral immune tolerance mechanisms. This breakdown allows the activation of myelin-based protein (MBP) specific CD8 lymphocytes, which cause the disease. Other hypotheses that the model takes into account are represented by a genetic predisposition of the host in developing the pathology, the presence of an external trigger that drives the starting the disease through antigen mimicry, and the effects caused by the malfunction (neural damage due to myelin loss). The model takes into account two populations representing specific CD8 lymphocytes that are the cause of neural damage and specific T regulatory lymphocytes, which may fail to suppress CD8 expansion. The model showed ability to capture the essential dynamics of relapsing-remitting MS by simulating both the absence and presence of malfunctions of the CD8-CD4-Treg crossbalancing mechanisms at a local level. In particular it suggested that the presence of a genetic predisposition is not always a sufficient condition for developing the disease. This model represents, up to now, the sole ABM model which models and reproduces the typical behavior of relapsing-remitting MS.

Several diseases like, for example, human immunodeficiency virus (HIV) or during some kind of medical interventions leave the immune system debilitated and not more able to fight properly against pathogens. Some opportunistic agents could invade the host with a damaged immune system and could lead to dangerous situations, and, in some cases, to the death of the patient. One class of these opportunist pathogens is represented by human pathogenic fungi like the ubiquitous fungus* Aspergillus fumigatus* that leads to mortality rates of 60–90% [38]. It is of great importance, then, to comprehend the infection dynamics and the response of the immune system to invading fungi: this clearly represents a crucial step on the way to stop the opportunistic pathogens from taking over.

Tokarski et al. [39] settled an ABM to investigate the influence of different tactics of neutrophils on their phagocytosis efficiency. The work focuses in particular on the question of whether chemical communication and chemotaxis of neutrophils improve the clearing efficiency. The authors used an important feature of NetLogo, that is, its capacity to run different simulations with different parameters settings. This characteristic is referred to as* behavior space*.

### 2.5. Tumor Immunology

The immune system is able to protect the host from tumor onset, and immune deficiencies are accompanied by an increased risk of cancer. The immune surveillance of tumors is not 100% effective. Tumors arise in hosts with a severe and stable immune deficiency. The study of tumor immunology has led to the development of approaches to further stimulate antitumor immunity. Immunological strategies for the cure of established tumor masses (immunotherapy) have given poor results suggesting that successful antitumor strategies should be addressed to adequately stimulate immune system before tumor onset (immunoprevention), to protect the organism from specific cancers, and to prevent tumor which is more effective than cure in the tumor immunology field [40].

In [41] the authors present a computer simulation model that uses an agent-based system developed with NetLogo to mimic the development and progression of solid tumors. The model includes tumor's own characteristics, the host immune response, and level of tumor vascularization. Simulations conducted indicate the key importance of the nutrient needs of the tumor cells and of the initial responsiveness of the immune system in the tumor progression.

Table 1 gives a brief overview of the NetLogo applications in immune system modeling.

## 3. Conclusions

In the last decades many mathematical and computational models have been developed to model and describe the immune system processes and features. Immune system is a complex biological system and has been recently investigated with the synergic union between computational modeling and high-throughput experimental data. There are several modeling techniques, each of them having both pros and cons. Among these, NetLogo may represent a mature choice for a number of reasons, spanning from its flexibility and its suitability for unskilled researchers and developers.

For multiscale or natural scale simulations the use of a high performance computing infrastructure, including machines and parallel code, is imperative. An evolution on this direction for such kind of tools would be important.

In this paper, we critically reviewed a large collection of works dealing with immune system modeling that successfully used NetLogo framework.

## Figures and Tables

**Table 1:** NetLogo applications in immune system modeling.

Modeling innate immunity	
Spatially configured stochastic reaction chambers (SCSRC)	[24]
Agent-based model of inflammation and fibrosis	[25]
Agent-based multiscale modular architecture for dynamic representation of acute inflammation	[26]
Agent-based modeling of endotoxin-induced acute inflammatory response in human blood leukocytes	[27]
Modeling immunity to pathogens	
Control of human papillomavirus	[28]
Model of human papillomavirus type 16	[29]
Intercellular peptide transfer through gap junctions	[30]
Connexin hemichannels enter the signalling limelight	[31]
Antigen transport and firebreaks In immune responses	[32]
Modeling immune system dynamic	
Mathematical epidemiology of infectious diseases	[33]
Heterogeneity in infection-exposure history and immunity of a protozoan parasite	[34]
Multicell agent-based simulation of the microvasculature	[35]
Modeling diseases	
Immunology of multiple sclerosis	[36]
Agent-based modeling of Treg-Teff cross regulation in relapsing-remitting multiple sclerosis	[37]
Molecular bases of virulence of *Candida albicans*, *Cryptococcus neoformans*, and *Aspergillus fumigates *	[38]
Agent-based modeling approach of immune defense against spores of opportunistic human pathogenic fungi	[39]
Tumor immunology	
Mathematical and computational models in tumor immunology	[40]
An agent-based model of solid tumor progression	[41]
